# Biomechanical Study of the Quadrupled Gracilis Tendon Graft for the Anterior Cruciate Ligament Reconstruction

**DOI:** 10.3390/jcm15218609

**Published:** 2026-11-05

**Authors:** Ivan Levaj, Ivan Bojanić, Ana Pilipović, Jan Homolak, Dinko Nizić, Marko Šimunović, Josip Vlaić, Mislav Jelić, Mario Josipović

**Affiliations:** 1 Department of Orthopedic Surgery, University Hospital Centre Zagreb, Šalata 7, 10000 Zagreb, Croatia; 2 Department of Orthopedic Surgery, School of Medicine, University of Zagreb, Šalata 7, 10000 Zagreb, Croatia; 3 Faculty of Mechanical Engineering and Naval Architecture, University of Zagreb, Ulica Ivana Lučića 5, 10000 Zagreb, Croatia; 4 Department of Pharmacology, School of Medicine, University of Zagreb, Šalata 11, 10000 Zagreb, Croatia; 5 Special Hospital Agram, Trnjanska Cesta 108, 10000 Zagreb, Croatia; 6 Special Hospital Medikol, Ulica Dragutina Mandla 7, 10000 Zagreb, Croatia; 7 Division of Paediatric Orthopaedic Surgery, Children’s Hospital Zagreb, Ulica Vjekoslava Klaića 16, 10000 Zagreb, Croatia

**Keywords:** all-inside ACL reconstruction, anterior cruciate ligament, biomechanics, gracilis tendon, hamstring autograft, semitendinosus tendon

## Abstract

**Background/Objectives**: To compare the morphological and biomechanical properties of a quadrupled gracilis (4GR) graft with two standard hamstring grafts for anterior cruciate ligament (ACL) reconstruction: a quadrupled semitendinosus (4ST) graft and a doubled semitendinosus–gracilis (2ST2GR) graft. **Methods**: Tendon specimens were obtained from 30 cadaveric donors (mean age, 42.6 years; range, 21–56 years). Tendon and graft dimensions were measured before biomechanical testing. Grafts underwent standardized preconditioning followed by load-to-failure testing at a displacement rate of 20 mm/min. Differences between graft types were analyzed using a generalized linear mixed-effects model, with results expressed as estimated marginal means with 95% confidence intervals (CI). **Results**: The mean 4GR graft length was 64.7 (95% CI, 61.6–67.8) mm, with 80% exceeding 60 mm. Graft diameter was greatest in the 4ST group, intermediate in the 2ST2GR group, and smallest in the 4GR group (7.98 [95% CI, 7.67–8.29], 7.41 [95% CI, 7.12–7.70], and 6.12 [95% CI, 5.89–6.36] mm, respectively). The 4ST graft demonstrated significantly greater maximum force than both the 4GR and 2ST2GR grafts, whereas no significant difference was observed between the 4GR and 2ST2GR grafts. In contrast, tensile strength was significantly higher in the 4GR grafts than in both the 4ST and 2ST2GR grafts. Stiffness was significantly higher in the 4ST grafts than in the 4GR grafts, whereas Young’s modulus was significantly higher in the 4GR grafts than in the 4ST grafts. **Conclusions**: The 4GR graft provided sufficient length for all-inside ACL reconstruction in most specimens. Despite its smaller diameter and lower absolute load-bearing capacity than 4ST, the 4GR graft demonstrated higher tensile strength and Young’s modulus, indicating favorable intrinsic mechanical properties. These findings may help inform graft selection in selected reconstructive procedures.

## 1. Introduction

Anterior cruciate ligament (ACL) rupture is one of the most common knee injuries requiring surgical treatment. Although ACL reconstruction is a well-established procedure that generally provides favorable clinical outcomes, each autograft option is associated with specific donor-site morbidity. The standard autograft options for ACL reconstruction include bone–patellar tendon–bone (BPTB), quadriceps tendon (QT), and hamstring tendon (HT) autografts. Among these, HT grafts are currently the most commonly used because they are associated with a lower incidence of anterior knee pain and less impairment of knee extensor strength than BPTB and QT grafts [1,2].

Advances in ACL reconstruction techniques have led to the development of the all-inside approach, a minimally invasive technique that uses retrograde drilling to create blind bone sockets instead of full-length bone tunnels [3]. A key technical advantage of the all-inside technique is its requirement for a substantially shorter graft, typically 60–70 mm in length [4,5]. Consequently, rather than harvesting both hamstring tendons as in conventional ACL reconstruction, a single tendon can be folded multiple times to create a graft with adequate diameter and biomechanical properties. Accordingly, isolated ST harvest has gained increasing popularity for all-inside ACL reconstruction [6,7].

Nevertheless, the hamstring muscles are important dynamic stabilizers of the knee, and isolated ST harvest is still associated with postoperative deficits in knee flexion strength, particularly at deeper flexion angles, and may impair tibial internal rotation [8,9,10]. Given the greater functional contribution of the ST muscle, preserving this tendon while using the GR as the sole graft source may represent a more favorable strategy. Previous studies have shown that isolated GR harvest is generally well tolerated and does not result in clinically meaningful impairment of knee or hip function [11,12].

Despite these potential advantages, ACL reconstruction using a gracilis-only graft has not yet been reported. This is likely attributable to the well-recognized anatomical characteristics of the GR tendon, which is generally shorter and smaller in diameter than the ST tendon [13]. The limited number of available cadaveric studies evaluating gracilis-only grafts have reported encouraging findings; however, the available evidence remains scarce and is characterized by important methodological limitations [14,15,16]. Therefore, the aim of the present study was to compare the anatomical and biomechanical properties of a quadrupled gracilis tendon graft (4GR) with two established four-strand hamstring graft configurations: a quadrupled semitendinosus graft (4ST) and a graft constructed from doubled semitendinosus and gracilis tendons (2ST2GR).

## 2. Materials and Methods

Tendon specimens were obtained from 30 cadaveric donors (23 males and 7 females; mean age, 42.6 years; range, 21–56 years). Ipsilateral GR and ST tendons were harvested from all donors, whereas an additional contralateral GR tendon was harvested from 15 donors, yielding a total of 45 GR and 30 ST tendons. The availability of contralateral GR tendons was determined by tissue availability, as other tendons had been allocated to separate research projects. These additional GR tendons enabled the preparation of 4GR grafts for both donor-matched experimental comparisons. Donors with evidence of tendon injury, previous lower-extremity surgery, or systemic disorders potentially affecting tendon quality were excluded. Anthropometric characteristics of the donors, including body height, anatomical and clinical leg length, thigh length, and thigh circumference, are presented in Supplementary Table S1.

All tendons were harvested within 24 h postmortem by an orthopedic surgeon using a standard surgical approach through a longitudinal skin incision over the pes anserinus insertion on the proximal tibia. A tendon stripper (Mitek Pigtail Tendon Peeler, DePuy Mitek, Raynham, MA, USA) was used to release the tendons proximally, after which residual muscle and adipose tissue were carefully removed, and the specimens were stored at −20 °C until testing. Before testing, all tendons were thawed overnight for approximately 12 h at room temperature in sealed packaging. Following thawing, excess surface moisture was removed using paper towels, and no additional hydration was applied during graft preparation or biomechanical testing to minimize the potential risk of graft slippage. Tendon mass was measured using an analytical balance (Radwag AS 220.R2 PLUS, Radwag, Radom, Poland; resolution, 0.0001 g). Tendon length was measured using a measuring tape, while width and thickness were measured using a digital caliper at four predefined anatomical locations: the distal insertion (1), the junction between the distal and middle thirds (2), the junction between the middle and proximal thirds (3), and the musculotendinous junction (4).

Following tendon measurements, three graft configurations were prepared: 4GR, 4ST, and 2ST2GR. A total of 30 4GR, 15 4ST, and 15 2ST2GR grafts were prepared and allocated into two experimental groups, each comprising 15 donor-matched pairs, with no overlap in donors between groups. In the first group, each 2ST2GR graft was prepared from GR and ST tendons harvested from one knee, whereas the corresponding 4GR graft was prepared from the GR tendon of the contralateral knee of the same donor. In the second group, 4GR and 4ST grafts were prepared from tendons harvested from the same knee. Donor allocation and graft distribution are illustrated in Supplementary Figure S1.

**Figure 1:**
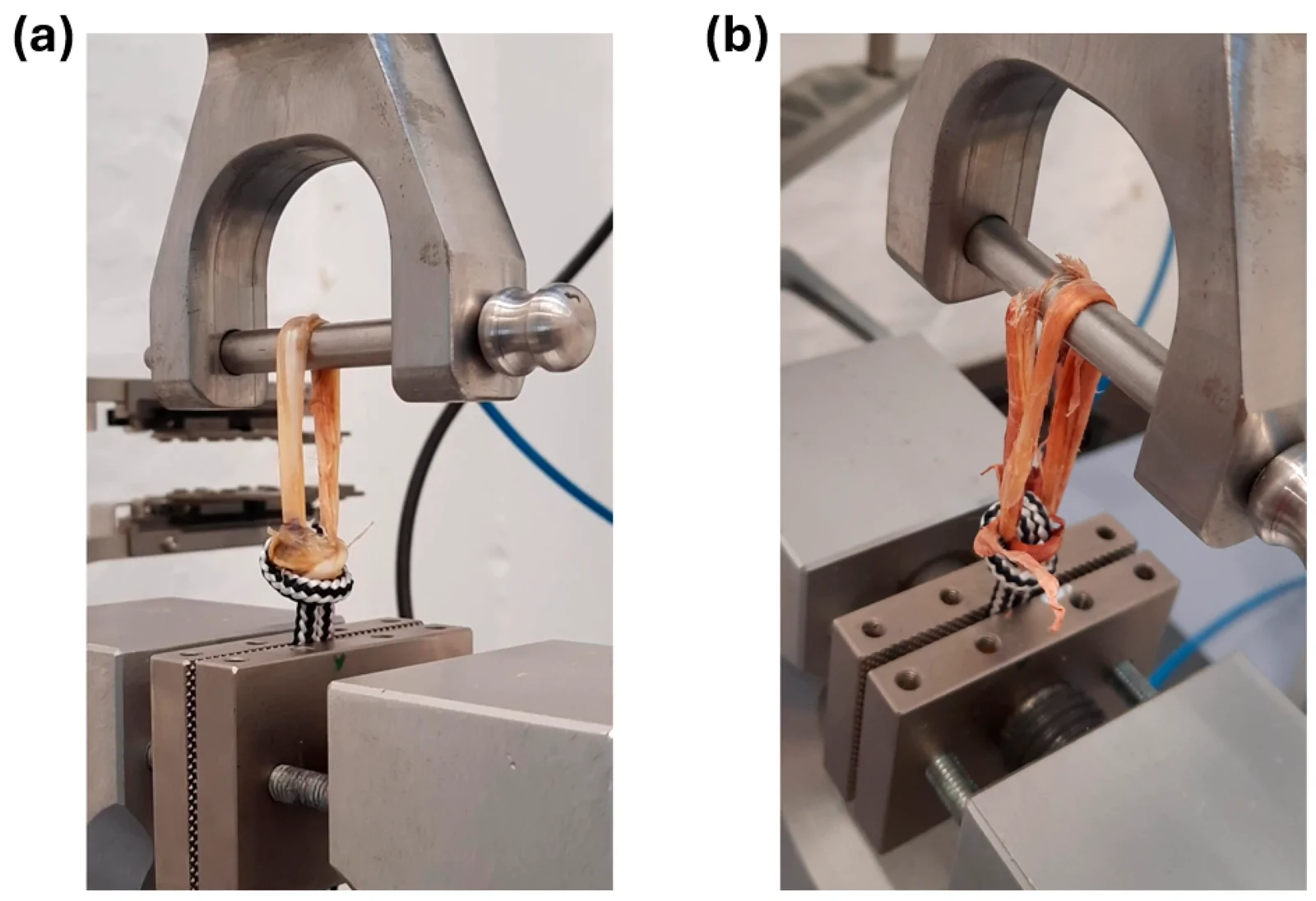
Photograph of the set-up showing a tendon graft positioned for biomechanical testing (**a**) and graft failure during load-to-failure testing (**b**).

Biomechanical testing was performed using a universal testing machine (Shimadzu AGS-X, Shimadzu Corporation, Kyoto, Japan; maximum load capacity, 10 kN) under controlled laboratory conditions (22 °C, 40% relative humidity). Grafts were mounted in a configuration simulating suspensory fixation, with the tendons folded over an 8-mm-diameter metal rod at one end. At the opposite end, the free tendon ends were secured using a self-tightening loop made of 4-mm-diameter polyester rope [17,18]. This loop was clamped between the grips of the testing machine, with a short segment remaining exposed between the grips and the tendon construct (Figure 1).

Before failure testing, all grafts underwent a preconditioning protocol consisting of a constant preload of 20 N applied for 60 s, followed by 10 loading cycles between 20 and 50 N at a loading rate of 6 N/s [19,20]. Subsequently, load-to-failure testing was performed at a constant displacement rate of 20 mm/min while force–displacement data were recorded continuously.

**Figure 2:**
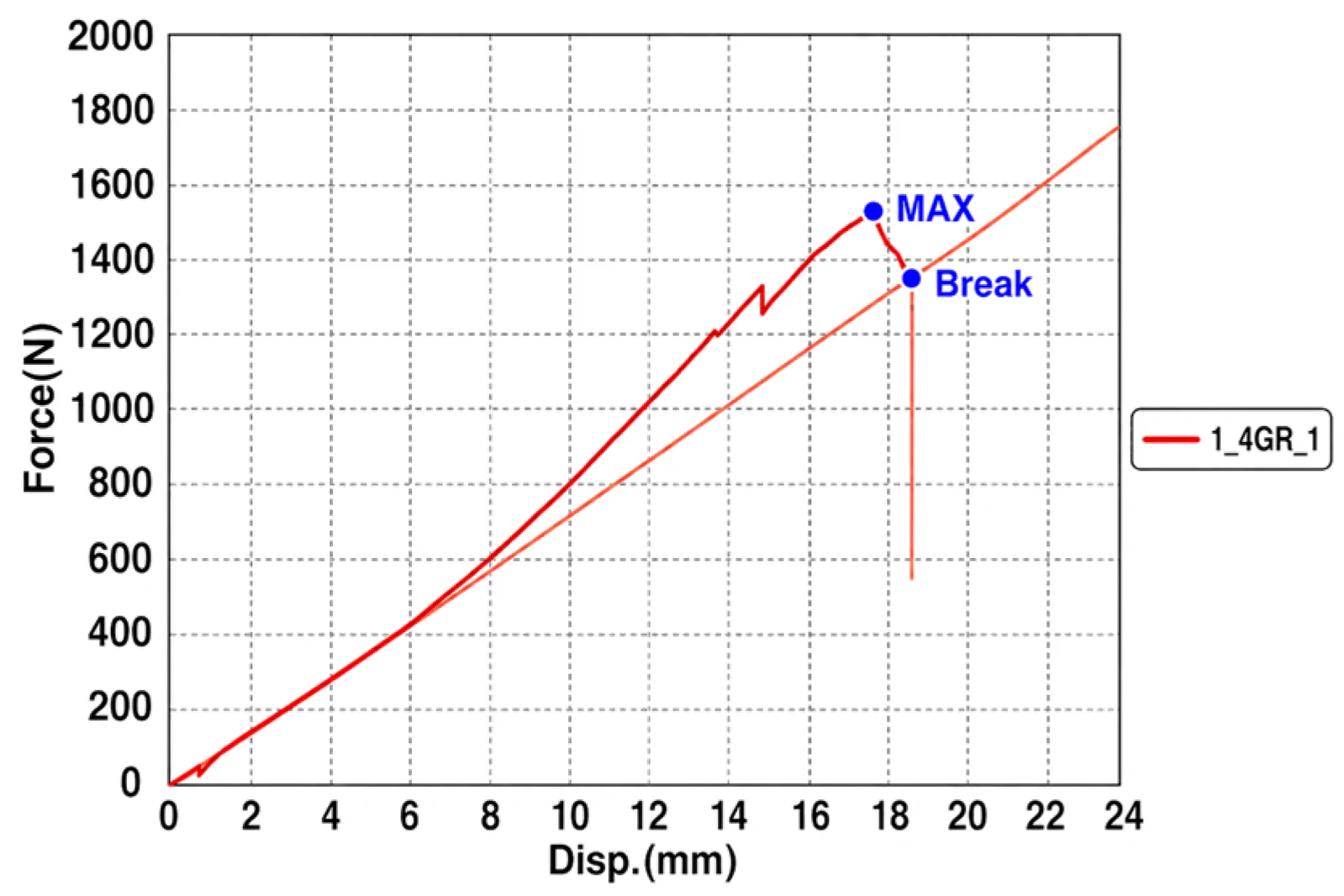
Representative force–displacement diagram of a 4GR graft during tensile testing, showing maximum force (MAX), failure force (Break), and the linear regression line used to determine graft stiffness.

Because tendon graft failure may occur through progressive fiber disruption rather than abrupt rupture, both maximum force and failure force were recorded. Maximum force was defined as the highest force sustained during testing and represents the onset of significant structural damage. Failure force was defined as the force at which functional graft failure occurred, identified by a pronounced loss of load-bearing capacity on the force–displacement curve (Figure 2). In cases of abrupt graft failure without a preceding decline in force, maximum force and failure force coincided.

**Figure 3:**
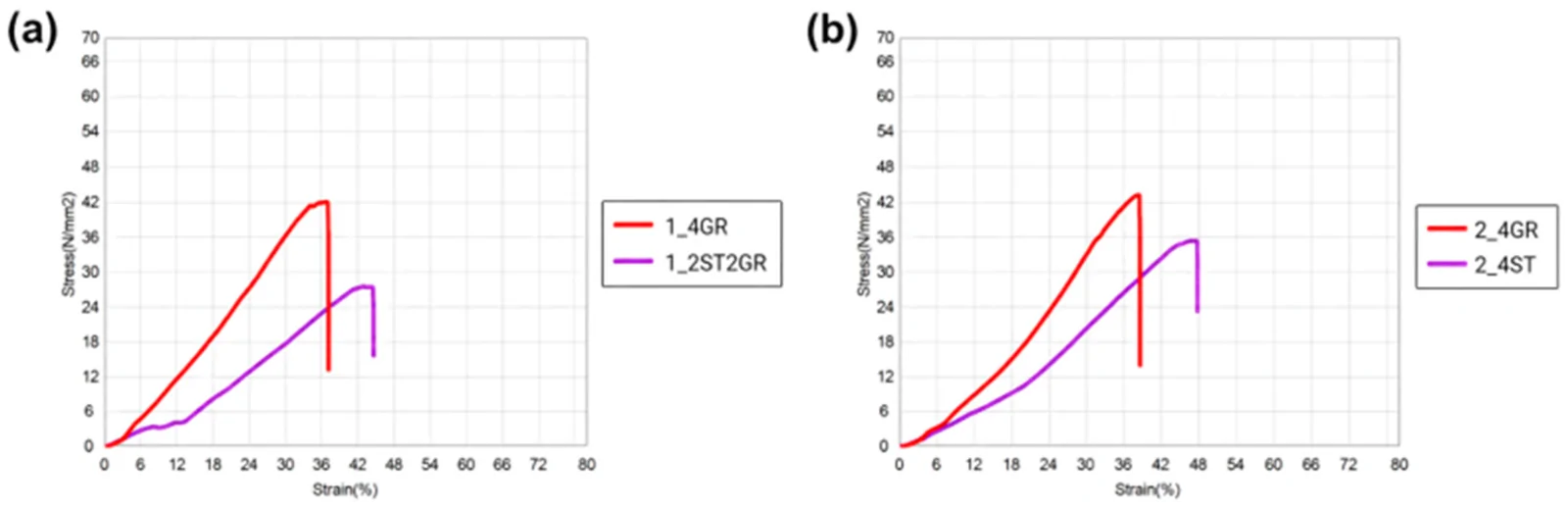
Representative stress–strain curves of donor-matched graft pairs during tensile testing, comparing 4GR with 2ST2GR (**a**) and 4GR with 4ST (**b**). The diagrams show mean value curves.

Stress–strain curves were derived from the force–displacement data (Figure 3). Tensile strength (*σ_max_*, N/mm^2^), corresponding to the maximum stress sustained by the graft during tensile loading, was calculated as:

(1) $$\sigma_{max}=\frac{F_{max}}{A_{0}}$$

where *F_max_* is the maximum force (N) and *A*_0_ is the initial graft cross-sectional area (mm^2^).

Failure strength (*σ_f_*, N/mm^2^), corresponding to the stress at functional graft failure, was calculated as:

(2) $$\sigma_{f}=\frac{F_{f}}{A_{0}}$$

where *F_f_* is the failure force (N).

The initial graft cross-sectional area (*A*_0_, mm^2^) was calculated from the measured graft diameter, assuming a circular cross-section:

(3) $$A_{0}=\frac{\pi d^{2}}{4}$$

where *d* is the measured graft diameter (mm).

Failure strain (*ε_f_*, %) represents the relative elongation of the graft at failure and was calculated as:

(4) $$\varepsilon_{f}=\frac{l_{f}-l_{0}}{l_{0}}\times 100$$

where *l*_0_ is the initial construct length (mm), and *l_f_* is the construct length at failure (mm).

Young’s modulus (*E*, N/mm^2^) was determined from the slope of the linear portion of the stress–strain curve within the stress range of 6–12 N/mm^2^ and was calculated as:

(5) $$E=\frac{\sigma_{2}-\sigma_{1}}{\varepsilon_{2}-\varepsilon_{1}}$$

where *σ*_1_ and *σ*_2_ are the stress values (N/mm^2^) and *ε*_1_ and *ε*_2_ are the corresponding strain values (mm/mm) within the specified stress range.

Energy absorption (J) and stiffness (*c*, N/mm) were automatically determined by the testing system. Energy absorption was calculated as the area under the force–displacement curve from the onset of loading to the point of maximum force, whereas stiffness was determined from the initial linear portion of the force–displacement curve.

### Statistical Analysis

Data were analyzed in R (v.4.3.1). The distribution of age was assessed using the Shapiro–Wilk test. Because the normality assumption was not met, sex differences in age were evaluated using the Wilcoxon rank-sum test. To account for age- and sex-related differences and the dependence of multiple specimens obtained from the same donor, morphometric and biomechanical outcomes were analyzed using generalized linear mixed-effects models (GLMMs) with the *lme4* and *emmeans* packages. Each outcome was modeled as a dependent variable, with age, sex, and tendon type (for morphometric outcomes) or graft type (for biomechanical outcomes), together with their interactions, included as fixed effects. Donor identity was included as a random intercept to account for within-donor correlation. Donor-specific random intercepts were assumed to follow a normal distribution with mean zero and an estimated variance. Observations were assumed to be conditionally independent given the donor random intercept and fixed effects, and observations from different donors were assumed to be independent. No additional residual correlation structure or random slopes were specified. Results are reported as model-derived estimated marginal means with 95% confidence intervals (CI). Pairwise comparisons between graft groups were performed with Holm adjustment for multiple comparisons. Model assumptions were checked by visual inspection of residuals. The significance level (α) was set at 0.05.

## 3. Results

**Figure 4:**
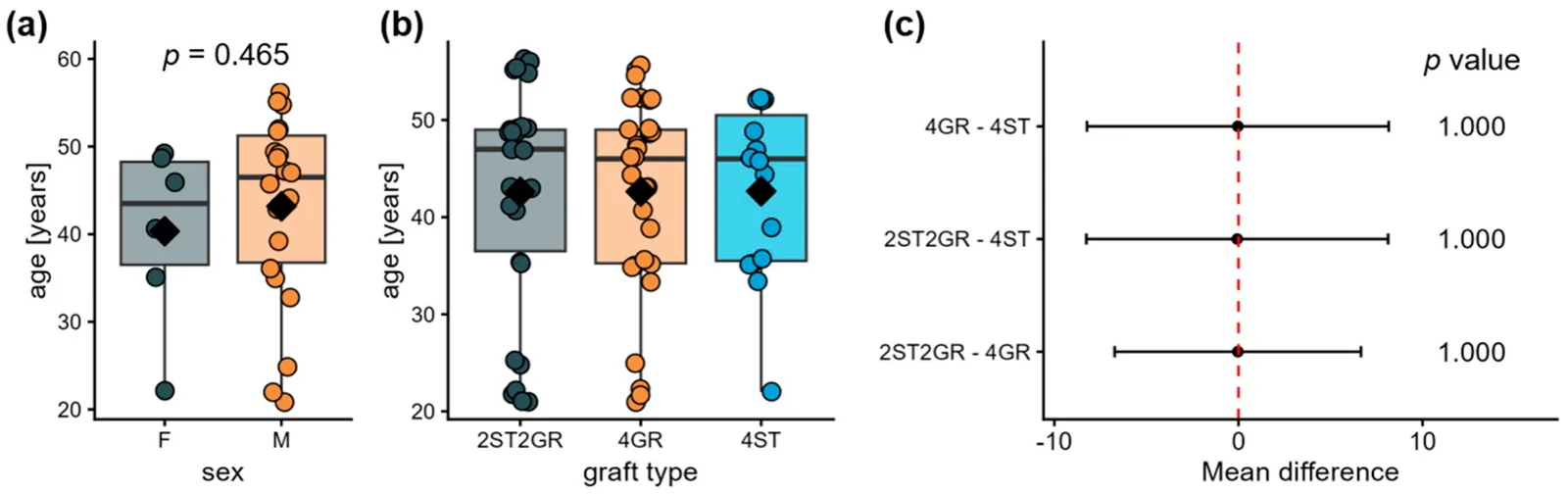
Donor age distribution according to sex (**a**) and graft type (**b**), with pairwise comparisons of mean donor age between graft-type groups based on a linear regression model (**c**). Panel (**c**) shows mean differences with 95% confidence intervals and corresponding *p*-values.

While all three graft configurations were not available as donor-matched specimens, no significant differences in donor age or sex distribution were observed between the two experimental groups, supporting their comparability (Figure 4). Therefore, morphometric and biomechanical outcomes were subsequently compared among 4GR, 4ST, and 2ST2GR grafts. Morphometric measurements were performed on all prepared grafts, whereas two grafts from the 2ST2GR group were excluded from the biomechanical analysis due to slippage from the gripping system during testing. Consequently, the final biomechanical analysis included 30 4GR, 15 4ST, and 13 2ST2GR grafts.

### 3.1. Tendon Morphology

The results of the morphometric analysis of ST and GR tendons are summarized in Table 1, with unadjusted descriptive statistics provided in Supplementary Table S2. ST tendons demonstrated significantly greater dimensions than GR tendons. They were significantly longer (288.95 [95% CI, 277.13–300.77] vs. 257.58 [95% CI, 246.30–268.86] mm; *p* < 0.0001), heavier (2.64 [95% CI, 2.43–2.84] vs. 1.22 [95% CI, 1.03–1.41] g; *p* < 0.0001), and exhibited greater width and thickness at nearly all measurement sites. The only exception was tendon width at the third measurement point, where no significant difference was observed (*p* = 0.9572).

Values are estimated marginal means (95% confidence intervals, CI) from a generalized linear mixed-effects model with tendon type, sex, age, and their interactions as fixed effects, and donor as a random effect. Pairwise mean differences and their 95% CI were estimated from the same statistical model and calculated as the first group minus the second, as indicated in the column headings. Negative values indicate lower estimates for the first group. Pairwise *p*-values were adjusted using the Holm method. Measurement sites 1–4 are defined in the Section 2.

### 3.2. Graft Morphology

Significant differences were observed in graft morphology among the three configurations. The 4ST grafts were significantly longer than 4GR grafts (71.9 [95% CI, 68.0–75.7] vs. 64.7 [95% CI, 61.6–67.8] mm; *p* = 0.0008). All 4ST grafts exceeded the minimum graft length of 60 mm required for all-inside ACL reconstruction, whereas this threshold was achieved by 80% of 4GR grafts. The length of 2ST2GR grafts was measured but was not included in the comparative analysis of graft length because this configuration is not intended for all-inside ACL reconstruction.

**Figure 5:**
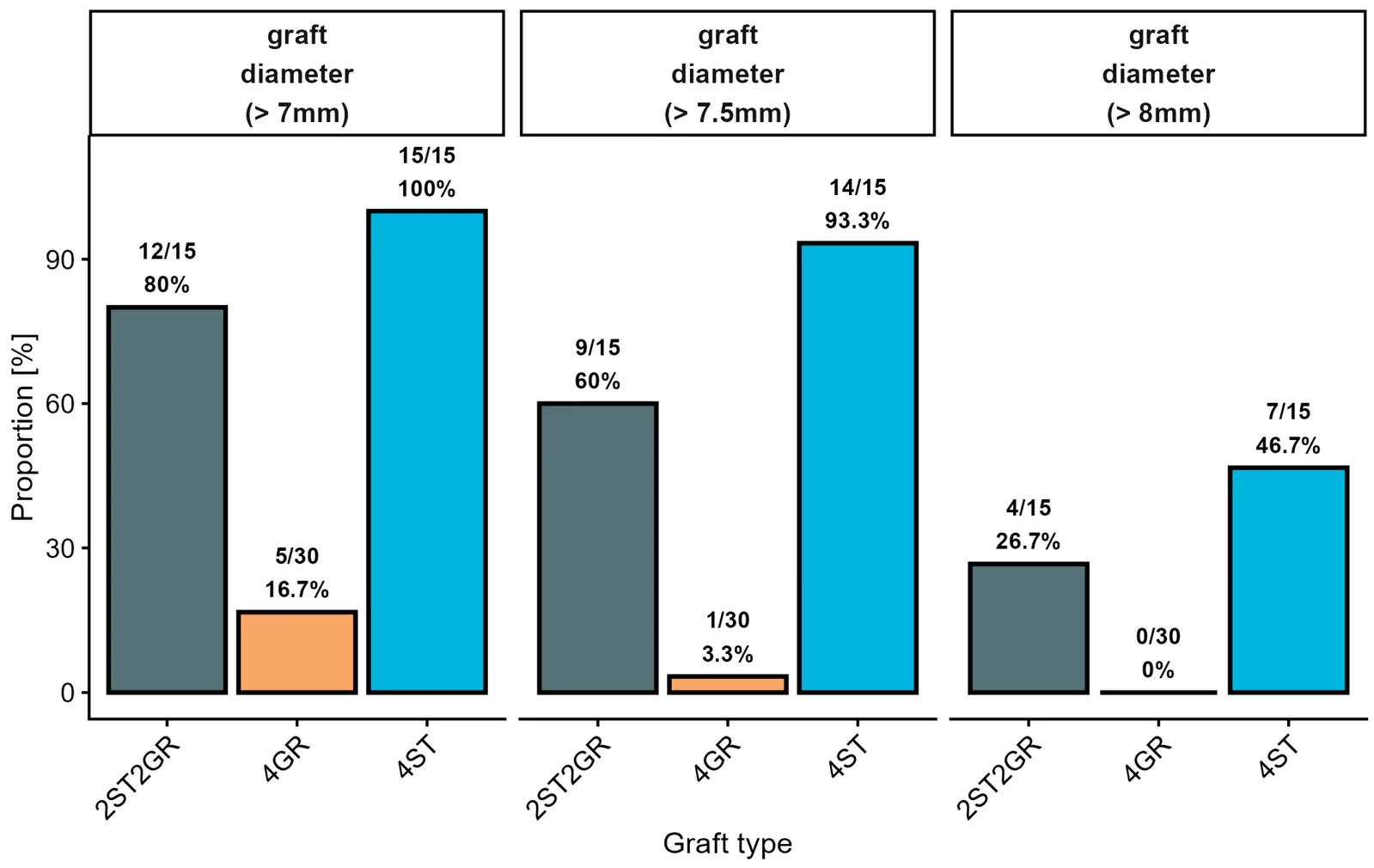
Proportion of grafts exceeding the predefined diameter thresholds according to graft type.

Graft diameter also differed significantly among the three configurations, with 4ST grafts demonstrating the largest estimated mean diameter (7.98 [95% CI, 7.67–8.29] mm), followed by 2ST2GR grafts (7.41 [95% CI, 7.12–7.70] mm) and 4GR grafts (6.12 [95% CI, 5.89–6.36] mm). All pairwise differences were significant (Holm-adjusted *p* < 0.01). All 4ST grafts exceeded 7 mm, compared with 80% of 2ST2GR grafts and only 16.7% of 4GR grafts. At thresholds of >7.5 mm and >8 mm, the proportion of grafts meeting these criteria progressively decreased, and none of the 4GR grafts exceeded 8 mm in diameter (Figure 5).

### 3.3. Biomechanical Properties

The results of the biomechanical analysis for all three graft configurations are summarized in Table 2, with unadjusted descriptive statistics provided in Supplementary Table S3. The 4ST grafts demonstrated significantly greater maximum force than both 4GR and 2ST2GR grafts (1980 [95% CI, 1740–2221] N vs. 1382 [95% CI, 1197–1568] N, *p* < 0.0001, and 1421 [95% CI, 1156–1686] N, *p* = 0.0041, respectively). Similarly, failure force was significantly greater in 4ST grafts (1922 [95% CI, 1657–2187] N) than in 4GR (1350 [95% CI, 1143–1557] N; *p* = 0.0004) and 2ST2GR grafts (1380 [95% CI, 1086–1673] N; *p* = 0.0116). No significant differences were observed between 4GR and 2ST2GR for either parameter.

In contrast, 4GR grafts demonstrated the highest tensile and failure strength. Tensile strength was significantly greater in 4GR than in both 2ST2GR (*p* = 0.0009) and 4ST grafts (*p* = 0.0358), while failure strength was also significantly greater than in both 2ST2GR (*p* = 0.0021) and 4ST grafts (*p* = 0.0450). No significant differences in tensile or failure strength were observed between 2ST2GR and 4ST grafts.

Failure strain was significantly greater in 4ST than in both 4GR and 2ST2GR grafts (both *p* = 0.0012), with no significant difference between 4GR and 2ST2GR grafts. Young’s modulus was significantly greater in 4GR than in 4ST grafts (*p* = 0.0182), while no significant differences were observed in the remaining pairwise comparisons. Energy absorption was greatest in 4ST grafts and significantly exceeded that of both 4GR (*p* < 0.0001) and 2ST2GR grafts (*p* = 0.0222). Stiffness was also highest in 4ST grafts and was significantly greater than that of 4GR grafts (*p* = 0.0082), while no significant differences were observed in the remaining pairwise comparisons.

Values are estimated marginal means (95% confidence intervals, CI) from a generalized linear mixed-effects model with graft type, sex, age, and their interactions as fixed effects, and donor as a random effect. Pairwise mean differences and their 95% CIs were estimated from the same statistical model and calculated as the first group minus the second, as indicated in the column headings. Negative values indicate lower estimates for the first group. Confidence intervals are Bonferroni-adjusted simultaneous intervals for the three pairwise comparisons, whereas *p*-values are Holm-adjusted for multiple testing.

## 4. Discussion

The findings of this study demonstrated that the GR tendon had significantly smaller dimensions than the ST tendon, including shorter length and lower mass, width, and thickness. Nevertheless, 4GR grafts provided sufficient length for all-inside ACL reconstruction in the majority of specimens, although their diameter was significantly smaller than that of both 4ST and 2ST2GR grafts. Biomechanically, 4GR grafts exhibited lower maximum and failure forces than 4ST grafts but showed comparable values to 2ST2GR grafts. Despite their lower absolute load-bearing capacity, 4GR grafts demonstrated the highest tensile and failure strength among the three graft configurations. Differences were also observed in failure strain, Young’s modulus, energy absorption, and stiffness, although the pattern of these differences varied among graft configurations.

The morphometric findings of the present study are consistent with previous anatomical studies demonstrating that the GR tendon is generally smaller than the ST tendon. Hamstring tendon length has been shown to correlate with body height and leg length, while significant differences in tendon length have also been reported between sexes [13,21,22]. Accordingly, reported mean GR tendon lengths range from 226.2 mm to 259.5 mm in females and from 239.7 mm to 299.0 mm in males. Similarly, mean ST tendon lengths range from 260.4 mm to 278.1 mm in females and from 267.3 mm to 318.4 mm in males [13,21,22,23]. These findings indicate considerable variability in absolute hamstring tendon dimensions across study populations, while consistently demonstrating greater length of the ST compared with the GR tendon [13,21,22,23].

Although the length of a quadrupled GR graft can be estimated from the length of the harvested tendon, only one previous study has directly evaluated 4GR graft length [15]. Cavaignac et al. reported that 4GR grafts exceeded the intra-articular length of the native ACL by at least 30 mm, with a mean difference of 38.9 mm, and concluded that 4GR grafts met the anatomical requirements for ACL reconstruction [15]. In the present study, all 4GR grafts were longer than 55 mm, with 80% reaching at least 60 mm, a length generally considered sufficient for all-inside ACL reconstruction.

Graft diameter is another important consideration in hamstring tendon ACL reconstruction, as smaller graft diameters have been associated with an increased risk of graft failure and revision [24,25,26]. However, no universally accepted minimum graft diameter has been established. Magnussen et al. proposed 8 mm as a clinically relevant threshold after demonstrating progressively higher revision rates with decreasing graft diameter: 1.7% for grafts >8 mm, 6.5% for grafts measuring 7.5–8 mm, and 13.6% for grafts ≤7 mm [24]. Spragg et al. subsequently demonstrated that, within the range of 7.0 to 9.0 mm, every 0.5 mm increase in graft diameter was associated with an 18% reduction in the odds of revision [25]. More recently, a systematic review and meta-analysis including 19,799 ACL reconstructions demonstrated a significantly lower failure rate for hamstring grafts ≥7 mm compared with those <7 mm [26]. In the present study, only 16.7% of 4GR grafts exceeded 7 mm in diameter, and none exceeded 8 mm. Similarly, Xie et al. reported a smaller mean diameter for 4GR than for 4ST grafts (5.9 ± 0.6 mm vs. 7.4 ± 0.7 mm), with only 8.1% of 4GR grafts measuring ≥7 mm and 1.3% measuring ≥7.5 mm [22].

However, graft diameter alone does not fully characterize the mechanical performance of a graft, and the clinical diameter thresholds established for conventional hamstring grafts may not be directly applicable to a 4GR construct. Biomechanical data specifically addressing the GR tendon and gracilis-only graft constructs remain limited [16,27,28,29,30,31]. Noyes et al. and Hamner et al. reported remarkably similar biomechanical properties for the isolated GR tendon, with mean maximum loads of 838 N and 837 N, stiffness values of 170 N/mm and 160 N/mm, and tensile strengths of 114 N/mm^2^ and 113.1 N/mm^2^, respectively [27,28]. Hamner et al. further demonstrated that doubling the GR tendon increased the maximum load to 1550 ± 428 N and stiffness to 336 ± 141 N/mm, corresponding to 185% and 210% of the respective single-strand values [28]. Handl et al. reported higher maximum loads of 925 ± 127 N for a single GR tendon and 2573 ± 496 N for a double-strand GR construct, with corresponding stiffness values of 354 and 432 N/mm [29]. To date, Pailhé et al. are the only authors to have specifically evaluated the biomechanical properties of 4GR grafts. They reported a substantially lower maximum load at failure of 416.4 ± 187.7 N and a stiffness of 192.85 ± 41.01 N/mm [16].

The considerable variability in absolute biomechanical values observed across the available literature, including the present findings, is likely attributable, at least in part, to differences in experimental methodology, particularly the loading rate used during tensile testing. Noyes et al. and Hamner et al. both used a strain rate of 100%/s and reported nearly identical maximum loads for the isolated GR tendon [27,28]. In contrast, Handl et al. employed a dynamic drop-weight testing system with impact velocities of up to 1.5 m/s [29], whereas Pailhé et al. used quasistatic tensile testing at a substantially slower crosshead speed of 10 mm/min following preloading and cyclic preconditioning [16]. Pailhé et al. considered these differences in loading conditions, together with the advanced age of their donors, as possible explanations for their markedly lower maximum load values [16].

Consequently, given the methodological variability between studies, comparisons between graft constructs tested under identical experimental conditions may be more informative than comparisons of absolute biomechanical values across studies. Pailhé et al. found that the 4GR construct had a significantly lower maximum load at failure than the 4ST construct (630.8 ± 239.1 N), while no significant differences were observed between 4GR and the 2ST2GR (473.5 ± 176.9 N) or patellar tendon grafts (413.3 ± 120.4 N) [16]. A similar pattern was observed in the present study, in which 4GR grafts demonstrated lower maximum and failure forces than 4ST grafts, whereas these values were comparable to those of the 2ST2GR construct. Regarding stiffness, Pailhé et al. reported the highest value for 4ST (249.78 ± 75.91 N/mm), followed by 2ST2GR (198.49 ± 44.92 N/mm) and 4GR (192.85 ± 41.01 N/mm), with only 4ST being significantly stiffer than the other graft constructs [16]. Similarly, in the present study, 4GR grafts demonstrated significantly lower stiffness than 4ST grafts, while no significant differences were observed between 4GR and 2ST2GR or between 4ST and 2ST2GR grafts.

Both maximum and failure forces, as well as stiffness, are influenced by graft dimensions and therefore do not fully reflect the intrinsic mechanical properties of the tendon tissue. When force was normalized to graft cross-sectional area, 4GR grafts demonstrated significantly higher tensile and failure strength than both 4ST and 2ST2GR grafts. To our knowledge, the present study is the first to report tensile strength specifically for a 4GR construct. Previous biomechanical studies evaluating single-strand tendons also reported higher tensile strength for GR than for ST. Hamner et al. reported values of 113.1 ± 18.1 N/mm^2^ for GR and 99.3 ± 14.9 N/mm^2^ for ST [28], while Handl et al. reported corresponding values of 95.1 ± 13.1 N/mm^2^ and 88.7 ± 7.8 N/mm^2^, respectively [29].

Young’s modulus provides a further measure of the intrinsic mechanical properties of the graft, as it is independent of graft dimensions. In the present study, 4GR grafts demonstrated a significantly higher Young’s modulus than 4ST grafts, whereas no significant difference was observed between 4GR and 2ST2GR grafts. Similar findings have been reported for single-strand tendons. Butler et al. reported a Young’s modulus of 612.8 ± 40.6 N/mm^2^ for GR compared with 362.2 ± 21.6 N/mm^2^ for ST [30], while Smeets et al. reported corresponding values of 1458 ± 476 N/mm^2^ and 1036 ± 312 N/mm^2^ [31].

Despite their favorable tensile strength and Young’s modulus, the relatively small diameter and lower absolute load-bearing capacity of 4GR grafts compared with 4ST grafts remain important limitations to their potential clinical application. Although preservation of the ST tendon may represent an advantage, the present findings do not establish whether this benefit outweighs the potential risks associated with smaller graft diameters. Furthermore, graft diameter thresholds associated with failure in conventional hamstring ACL reconstruction cannot necessarily be extrapolated to 4GR grafts. Therefore, the clinical role of 4GR grafts remains uncertain and requires further investigation before their use can be recommended.

This study has several limitations. First, all three graft configurations could not be prepared from the same donor because bilateral GR and ST tendon specimens were not available. Although comparability between the paired graft groups was confirmed, a fully within-donor comparison of all three graft configurations would have provided better control of interindividual variability. Second, the polyester fixation loop was subjected to tensile loading together with the tendon graft and may have contributed to the recorded displacement, potentially influencing displacement-dependent biomechanical parameters, including failure strain, stiffness, Young’s modulus, and energy absorption. However, only a short segment of the polyester loop was exposed during testing, and the fixation configuration, loop material, and exposed loop length were standardized across all graft groups. Therefore, although the contribution of the fixation loop could not be quantified separately, the consistent testing conditions support comparisons between the investigated graft configurations. Furthermore, the native ACL and other commonly used ACL grafts, such as BPTB and QT grafts, were not included in the experimental protocol. Their inclusion would have allowed a direct comparison of the biomechanical properties of the 4GR construct with the native ligament and a broader range of established graft options under identical experimental conditions. Finally, as a time-zero cadaveric biomechanical study, the present investigation cannot reproduce the biological incorporation and remodeling of the graft or fully reflect its mechanical behavior in vivo.

## 5. Conclusions

The 4GR graft demonstrated sufficient length for all-inside ACL reconstruction in the majority of specimens, along with favorable intrinsic mechanical properties, particularly tensile strength and Young’s modulus. However, its relatively small diameter, together with lower maximum force and stiffness compared with the 4ST graft, raises concerns regarding its clinical applicability in ACL reconstruction. Further investigation is therefore warranted to establish the potential role of the 4GR construct in all-inside ACL reconstruction, particularly when preservation of the ST tendon is desired.

## Figures and Tables

**Table 1:** Morphometric characteristics of gracilis (GR) and semitendinosus (ST) tendons and between-group comparisons.

	GR Tendons (*n* = 45)	ST Tendons (*n* = 30)	Estimated Mean Difference GR–ST	*p*
Tendon length (mm)	257.58 (246.30–268.86)	288.95 (277.13–300.77)	−31.37 (−40.90–−21.84)	<0.0001
Tendon mass (g)	1.22 (1.03–1.41)	2.64 (2.43–2.84)	−1.42 (−1.61–−1.23)	<0.0001
Tendon width 1 (mm)	5.32 (4.85–5.79)	5.95 (5.42–6.47)	−0.63 (−1.24–−0.02)	0.0438
Tendon width 2 (mm)	3.42 (3.02–3.82)	4.15 (3.72–4.58)	−0.73 (−1.16–−0.29)	0.0015
Tendon width 3 (mm)	4.59 (4.05–5.12)	4.57 (3.99–5.15)	−0.02 (−0.57–0.60)	0.9572
Tendon width 4 (mm)	4.01 (3.37–4.64)	6.71 (6.00–7.42)	−2.71 (−3.55–−1.87)	<0.0001
Tendon thickness 1 (mm)	1.56 (1.41–1.71)	1.78 (1.61–1.95)	−0.22 (−0.44–−0.01)	0.0451
Tendon thickness 2 (mm)	1.80 (1.67–1.93)	2.50 (2.35–2.65)	−0.7 (−0.86–−0.54)	<0.0001
Tendon thickness 3 (mm)	1.87 (1.67–2.07)	2.80 (2.58–3.01)	−0.93 (−1.17–−0.68)	<0.0001
Tendon thickness 4 (mm)	1.33 (1.14–1.52)	1.95 (1.73–2.18)	−0.63 (−0.92–−0.33)	<0.0001

**Table 2:** Biomechanical properties and pairwise comparisons of different graft configurations: quadrupled gracilis (4GR), quadrupled semitendinosus (4ST), and doubled semitendinosus–gracilis (2ST2GR) grafts.

Biomechanical Parameter	2ST2GR (*n* = 13)	4GR (*n* = 30)	4ST (*n* = 15)	Estimated Mean Difference 2ST2GR–4GR	Estimated Mean Difference 4GR–4ST	Estimated Mean Difference 2ST2GR–4ST	*p* 2ST2GR vs. 4GR	*p* 4GR vs. 4ST	*p* 2ST2GR vs. 4ST
Maximum force (N)	1421 (1156–1686)	1382 (1197–1568)	1980 (1740–2221)	39.0 (−307.2–385.3)	−598.2 (−911.5–−285.0)	−559.2 (−986.0–−132.4)	0.7819	<0.0001	0.0041
Tensile strength (N/mm^2^)	33.5 (26.7–40.2)	47.5 (42.8–52.2)	39.4 (33.3–45.6)	−14.1 (−23.0–−5.1)	8.1 (−0.1–16.3)	−6.0 (−17.0–5.1)	0.0009	0.0358	0.1877
Failure force (N)	1380 (1086–1673)	1350 (1143–1557)	1922 (1657–2187)	29.7 (−346.7–406.0)	−571.5 (−909.0–−234.0)	−541.9 (−1008.1–−75.7)	0.8465	0.0004	0.0116
Failure strength (N/mm^2^)	32.4 (24.8–39.9)	46.5 (41.2–51.8)	38.2 (31.4–45.1)	−14.1 (−23.8–−4.4)	8.3 (−0.4–16.9)	−5.9 (−17.9–6.2)	0.0021	0.0450	0.2342
Failure strain (%)	33.8 (28.8–38.8)	37.3 (33.7–40.8)	45.6 (41.1–50.1)	−3.5 (−9.7–2.7)	−8.3 (−13.8–−2.8)	−11.8 (−19.5–−4.1)	0.1703	0.0012	0.0012
Young’s modulus (N/mm^2^)	114.2 (96.0–132.0)	111.6 (97.9–125)	90.8 (74.3–107)	2.6 (−18.2–23.5)	20.9 (2.9–38.9)	23.5 (−2.9–49.8)	0.7564	0.0182	0.0638
Energy absorption (J)	14.0 (11.1–17.0)	9.9 (7.8–12.0)	18.5 (15.8–21.2)	4.2 (0.4–7.9)	−8.6 (−12.0–−5.3)	−4.5 (−9.2–0.2)	0.0181	<0.0001	0.0222
Stiffness (N/mm)	79.6 (70.0–89.3)	72.7 (66.0–79.5)	87.3 (78.5–96.1)	6.9 (−5.7–19.5)	−14.5 (−25.9–−3.2)	−7.7 (−23.2–7.9)	0.3664	0.0082	0.3664

## Data Availability

Data are contained within the article.

## Supplementary Materials

The following supporting information can be downloaded at: https://www.mdpi.com/article/10.3390/jcm15218609/s1, Figure S1: Donor allocation and graft distribution across the two experimental groups; Table S1: Anthropometric characteristics of cadaveric donors according to sex; Table S2: Unadjusted descriptive summary statistics for morphometric characteristics of tendons; Table S3: Unadjusted descriptive summary statistics for biomechanical properties of grafts.

## Abbreviations

The following abbreviations are used in this manuscript:

ACL	Anterior cruciate ligament
BPTB	Bone–patellar tendon–bone
QT	Quadriceps tendon
HT	Hamstring tendon
ST	Semitendinosus
GR	Gracilis
4GR	Quadrupled gracilis graft
4ST	Quadrupled semitendinosus graft
2ST2GR	Double-stranded semitendinosus and gracilis graft
