# Supplementary material for: Biomechanical Study of the Quadrupled Gracilis Tendon Graft for the Anterior Cruciate Ligament Reconstruction
Source: J Clin Med. 2026 Nov 5;15(21):8609. doi: 10.3390/jcm15218609 (PMC13764330; doi:10.3390/jcm15218609)
Supplement: Supplementary file 1 [file jcm-15-08609-s001.zip › Supplementary Figure S1.pdf]

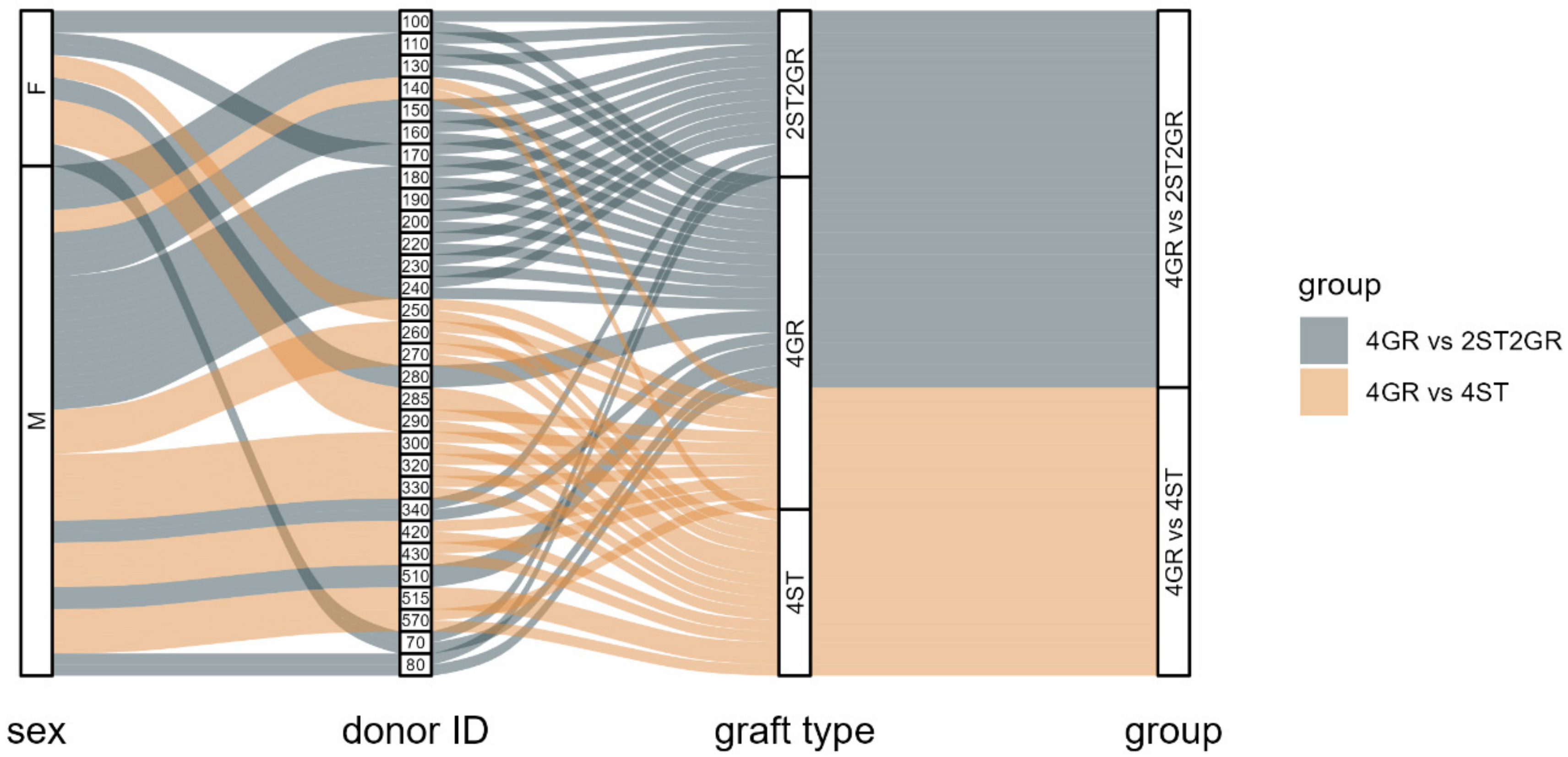

Supplementary Figure S1. Alluvial diagram illustrating donor allocation and graft distribution across the two experimental groups. F, female; M, male; 4GR, quadrupled gracilis tendon graft; 4ST, quadrupled semitendinosus tendon graft; 2ST2GR, doubled semitendinosus–gracilis tendon graft.
